# In Situ Monitoring of Mechanofluorescence in Polymeric Nanofibers

**DOI:** 10.1002/marc.202400855

**Published:** 2024-12-23

**Authors:** Valentina A. Dini, Derek J. Kiebala, Damiano Genovese, Nelsi Zaccheroni, Céline Calvino, Emma Contini, Christoph Weder, Stephen Schrettl, Chiara Gualandi

**Affiliations:** ^1^ Department of Chemistry “Giacomo Ciamician” University of Bologna Via Selmi 2 Bologna 40126 Italy; ^2^ Adolphe Merkle Institute (AMI) Polymer Chemistry and Materials University of Fribourg Chemin des Verdiers 4 Fribourg CH‐1700 Switzerland; ^3^ National Competence Center in Research Bio‐inspired Materials University of Fribourg Chemin des Verdiers 4 Fribourg CH‐1700 Switzerland; ^4^ Department of Chemistry Johannes Gutenberg University of Mainz 55128 Mainz Germany; ^5^ INSTM UdR of Bologna University of Bologna Via Selmi 2 Bologna 40126 Italy; ^6^ TUM School of Life Sciences Technical University of Munich Maximus‐von‐Imhof‐Forum 2 85354 Freising Germany; ^7^ Interdepartmental Center for Industrial Research on Advanced Applications in Mechanical Engineering and Materials Technology CIRI‐MAM University of Bologna Viale Risorgimento, 2 Bologna 40136 Italy

**Keywords:** electrospinning, mechanochromism, mechanofluorescent materials, nanofibers, strain sensing

## Abstract

Mechanofluorescent polymers represent a promising class of materials exhibiting fluorescence changes in response to mechanical stimuli. One approach to fabricating these polymers involves incorporating aggregachromic dyes, whose emission properties are governed by the intermolecular distance, which can, in turn, be readily altered by microstructural changes in the surrounding polymer matrix during mechanical deformation. In this study, a mechanofluorescent additive featuring excimer‐forming oligo(*p*‐phenylene vinylene) dyes (tOPV) is incorporated into electrospun polyurethane fibers, producing mats of fibers with diameters ranging from 300 to 700 nm. The influence of the additive concentration and fiber orientation on the mechanofluorescent response under tensile deformation is investigated. In situ fluorescence spectroscopy and microscopy imaging reveal a strain‐dependent change of the fluorescence color from orange to yellow or green, with a more pronounced response in prealigned fibers. Stresses experienced by the nanofibers during elongation are mapped in real‐time. The data reveal that forces initially concentrate in fibers that are aligned parallel to the applied strain, and only later redistribute as other fibers once they also align. These findings advance the understanding of force transfer within fibrous polymer mats and are expected to facilitate the development of self‐reporting nanofibers for applications in load‐bearing devices, wearable technologies, and mechanochromic textiles.

## Graphical abstract

Mechanochromic polyurethane fibers containing a mechanofluorescent additive are produced. The influence of additive concentration and fiber orientation on mechanochromism under tensile deformation is elucidated using in situ fluorescence spectroscopy and microscopy. A strain‐dependent fluorescence color change is observed, with a more pronounced response in prealigned fibers. These results improve understanding of force transfer in fibrous mats, aiding the development of self‐reporting nanofibers.

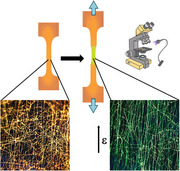

## 1 Introduction

Polymeric fibers enable a wide range of applications, including drug delivery, diagnostics, tissue engineering, “smart” optical systems, biosensors, coatings, and textiles. Monitoring the structural integrity of fibrous materials during their use is critical to ensuring their performance and preventing premature failure. However, accurate and non‐invasive methods for such monitoring are currently lacking. Addressing this challenge by endowing polymeric fibers with the ability to transform external stimuli such as mechanical stress or temperature variations, into readily measurable signals could provide valuable real‐time information on the fibers’ morphology and environment without the need for invasive probes.^[^ ^1^ ^−^ ^4^ ^]^

Recently, some of us reported a mechanochromic additive based on a telechelic macromolecule carrying excimer‐forming oligo(*p*‐phenylene vinylene) dyes at its termini (tOPV).^[^ ^5^ ^−^ ^7^ ^]^ This sensor molecule was shown to microphase‐separate when blended in small quantities with different host polymers (typically between 0.2 and 1 wt %). The spherical, Eshelby‐type inclusions that form in this process deform homogeneously and reversibly in response to macroscopically applied mechanical forces.^[^ ^7^ ^]^ This results in a fluorescence color change that is directly proportional to the applied strain and reversible over multiple deformation‐relaxation cycles. Crucially, the mechanochromism is observed in both semicrystalline and amorphous polymers–unlike many polymers with small‐molecule additives, whose mechanical activation requires significant shear forces.^[^ ^5^, ^6^ ^]^ Given the tOPV additive's compatibility with various processing conditions, we sought to explore its potential in nanostructured materials. Specifically, we hypothesized that blending tOPV with polymeric nanofibers could yield mechanofluorescent fibers that are uniquely suited for high‐precision strain sensing applications.^[^ ^8^ ^−^ ^14^ ^]^ Methods reported thus far for producing mechanochromic fibrous materials include dip‐coating commercially available yarns with core‐shell microspheres or colloidal crystals,^[^ ^8^, ^9^ ^]^ and the fabrication of cholesteric liquid crystal elastomer filaments that exhibit a progressive and reversible mechanochromic response.^[^ ^14^ ^]^ Moreover, molecular mechanophores have been covalently incorporated into the fiber's constituent polymers; fibers from spiropyran‐functionalized polymers exhibit a strain‐induced mechanochromic response upon transition from the colorless spiropyran to the purple merocyanine form.^[^ ^10^, ^11^ ^]^

Various methods are available for producing polymer nanofibers, such as electrophoretic deposition, centrifugal spinning, and electrospinning. Among those, electrospinning is particularly attractive, since it allows to precisely control the fiber morphology and alignment by adjusting basic parameters such as the composition of the polymer solution (e.g., molecular weight, concentration, electrical conductivity, viscosity), processing conditions (e.g., applied voltage, tip‐to‐collector distance, flow rate), and environmental factors (e.g, temperature, relative humidity).^[^ ^15^, ^16^ ^]^ This versatility has enabled the application of electrospun nanofibrous materials in fields such as water remediation,^[^ ^17^ ^−^ ^21^ ^]^ catalysis,^[^ ^22^, ^23^ ^]^ energy harvesting and storage,^[^ ^24^ ^−^ ^27^ ^]^ structural composites,^[^ ^28^, ^29^ ^]^ and biomedical technologies.^[^ ^30^ ^−^ ^32^ ^]^

**Figure 1:**
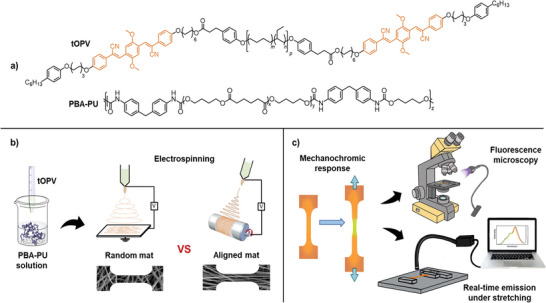
a) Chemical structure of the excimer‐forming tOPV additive (with *M*_n_ = 4500 g mol^−1^; *m* ≈ 0.36, *n* ≈ 0.64, *p* ≈ 44) and the poly(butyl adipate)‐containing polyurethane (PBA‐PU) used in this study. b) Schematic representation of the fabrication process of electrospun mats comprised of either randomly oriented or aligned fibers. c) Schematic of the experimental framework used to characterize the mechanochromic response of the fiber mats under tensile deformation, which includes real‐time emission detection and fluorescence microscopy.

To elucidate the structure‐property relationships that govern the mechanical behavior of these versatile materials, we herein report the fabrication of mechanofluorescent fibers by electrospinning polyurethane solutions containing small amounts of the tOPV additive (**Figure** **1**). The resulting electrospun mats consist of fibers that are either randomly oriented or uniaxially aligned, depending on the type of collector used during the electrospinning process (Figure 1). We demonstrate that both types of fiber mats exhibit mechanochromic behavior under mechanical strain and that the specific response to the applied strain depends on the degree of fiber orientation. Fluorescence microscopy analysis of individual fibers within the mats reveals that fibers aligned along the strain direction deform more significantly than those oriented perpendicularly. These findings provide valuable insights into the force transfer within complex fibrous mats, enabling the rational design of electrospun fiber mats with tailored mechanical properties for a wide array of potential applications.

## 2 Results and Discussion

**Figure 2:**
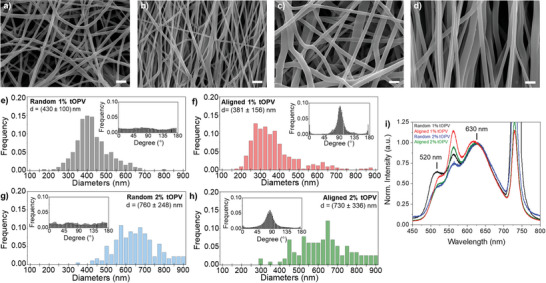
a–d) SEM images of electrospun mats composed of randomly oriented (a, c) and uniaxially aligned (b, d) fibers containing 1 wt% (a, b) and 2 wt% (c, d) of the tOPV additive (scale bar: 2 µm). e–h) Histograms showing the diameters of the fibers pictured in (a–d), with insets showing the analysis of the distribution of fiber orientations. i) Comparison of the normalized fluorescence spectra of electrospun fibers with varying tOPV concentrations and fiber orientations. Spectra are normalized to the excimer emission band at 630 nm. The symmetric band at 730 nm is due to the excitation beam (365 nm) passing through the grating at twice the wavelength.

Poly(butyl adipate)‐containing polyurethane (PBA‐PU) is a commercially available semicrystalline poly(ester urethane) that has been widely studied for its shape memory properties.^[^ ^33^ ^−^ ^35^ ^]^ This material combines the high deformability of polyurethanes with the plastic‐like behavior of the semicrystalline PBA.^[^ ^36^ ^]^ In this context, the crystallizability of PBA imparts the material with sufficient structural integrity to preserve fiber morphology during solvent evaporation, while maintaining its high deformability. Electrospinning PBA‐PU solutions with either 1 or 2 wt% tOPV yielded homogeneous, bead‐free nanofiber mats (**Figure** **2**a–d). The PBA‐PU/tOPV fibers containing 1 wt% tOPV exhibited a mean diameter of ca. 400 nm, while those blended with 2 wt% tOPV display a larger mean diameter of ca. 700 nm (Figure 2e–h). This increase in fiber diameter with higher tOPV content is consistent with the higher polymer concentration in the electrospinning solutions,^[^ ^37^, ^38^ ^]^ although the magnitude of the effect suggests additional factors may be involved. Scanning electron microscopy (SEM) revealed that fibers electrospun on a flat collector were randomly oriented, whereas those electrospun on a rotating drum were primarily aligned along the wind‐up direction (Figure 2e‐h, insets). During electrospinning, the mechanophore's emission was monitored under UV light (365 nm, Video S1, Supporting Information). The visual observation revealed that the PBA‐PU spinning solutions containing the tOPV emit green light upon ejection from the nozzle, while the fibers deposited on the flat collector appeared orange. These different emission colors are associated with monomer (green) and excimer (orange) emission from the tOPV's terminal oligo(*p*‐phenylene vinylene) dyes,^[^ ^39^, ^40^ ^]^ which suggests that the fast solvent evaporation during electrospinning causes the dyes to aggregate as the fibers form.^[^ ^7^ ^]^

The emission spectra of both randomly oriented and aligned tOPV‐blended fiber mats display the characteristic tOPV monomer (*λ*_max_ = 520 nm) and excimer (*λ*_max_ = 630 nm) emission bands (Figure 2i), with excimer emission intensity dominating. As previously reported, the ratio of the tOPV monomer emission intensity (*I_M_* at 520 nm) to the corresponding excimer emission intensity (*I_E_* at 630 nm), referred to as *I*_M_/*I*_E_, is a convenient measure to express the emission color of tOPV.^[^ ^5^ ^−^ ^7^ ^]^ For the PBA‐PU/tOPV 1 wt% mats, *I*_M_/*I*_E_ shows values of approximately 0.65 (Table S1, Supporting Information), higher than previously reported values for the same blend in bulk films (ca. 0.4).^[^ ^6^ ^]^ This suggests that electrospinning reduces the extent of tOPV aggregation, perhaps due to the kinetic trapping of some tOPV macromolecules during rapid solvent evaporation and fiber solidification. Moreover, increasing the tOPV concentration in the fibers not only led to the formation of fibers with increased diameters but also decreased *I*_M_ */I*_E_ values, indicating a higher fraction of dye aggregates (Table S1, Supporting Information). An additional emission band at *λ*_max_ = 562 nm, which can be attributed to the formation of “Y‐form” OPV excimers,^[^ ^41^ ^]^ was observed in all electrospun samples (Figure 2i), suggesting that this excimer form is promoted by the high shear rates (100–1000 s^−1^) and draw ratios (up to 25 000) characteristic of the electrospinning process.^[^ ^42^, ^43^ ^]^

**Figure 3:**
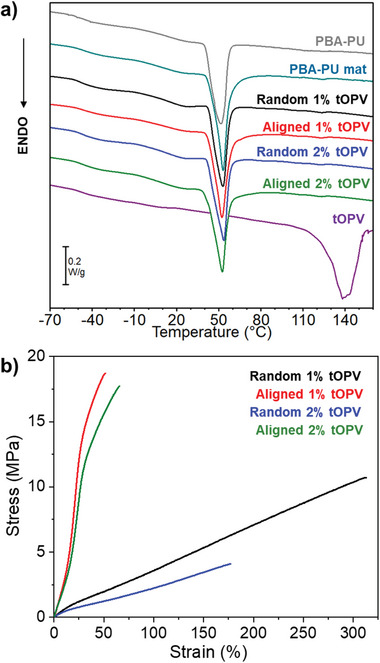
a) DSC first heating scans of bulk PBA‐PU (grey), electrospun PBA‐PU (teal), random PBA‐PU/tOPV 1 wt% (black), aligned PBA‐PU/tOPV 1 wt% (red), random PBA‐PU/tOPV 2 wt% (blue), aligned PBA‐PU/tOPV 2 wt% (green), and tOPV (purple) fiber mats. b) Representative stress‐strain curves from uniaxial tensile testing (strain rate = 30% min^−1^) of random PBA‐PU/tOPV 1 wt% (black), aligned PBA‐PU/tOPV 1 wt% (red), random PBA‐PU/tOPV 2 wt% (blue), and aligned PBA‐PU/tOPV 2 wt% (green) fiber mats.

Differential scanning calorimetry (DSC) measurements revealed that the fiber mats’ glass transition temperature (*T*_g_) at ca. –46 °C matches that of bulk PBA‐PU, i.e., well below room temperature (**Figure** **3**a and Table S1, Supporting Information). The DSC traces further indicate the presence of a crystalline PBA phase with a melting temperature (*T*_m_) of 50 °C. Notably, both randomly oriented and aligned fiber mats made with the same tOPV concentration exhibit similar *T*_g_ and *T*_m_ values (Table S1, Supporting Information). However, the aligned fibers show slightly higher melting enthalpies than the randomly oriented ones, suggesting a higher degree of crystallinity in the former (Table S1, Supporting Information). This increased crystallinity can be attributed to the additional stretching of the macromolecules during collection on the high‐velocity rotating drum, which promotes polymer crystallization by enhancing chain alignment along the fiber axis, as previously reported for other polymers.^[^ ^44^, ^45^ ^]^

The fiber mats were subjected to uniaxial tensile deformation to probe the influence of fiber orientation on their mechanical properties (Figure 3b, Figure S1 and Table S2, Supporting Information). When comparing PBA‐PU fibers without the additive with the corresponding samples containing tOPV, it becomes apparent that the addition of the telechelic dye decreases the strain at break while the Young's modulus and the stress at break remain almost unaffected. When measured along the orientation direction, aligned fiber mats exhibit a significantly higher stiffness and stress at break compared to the randomly oriented mats, along with a consistently lower strain at break (Figure 3b; Figure S1, Supporting Information). While the Young's modulus of the aligned mats was four times lower than that of bulk PBA‐PU (Table S2, Supporting Information), the increase in mechanical stress during strain hardening was significantly steeper for the mats than for bulk films (Figure 3b).^[^ ^6^ ^]^ Specifically, the aligned mats reached a maximum stress of ≈18 MPa between 35 and 50% strain, whereas bulk PBA‐PU experienced only around 8 MPa stress at 50% strain.^[^ ^6^ ^]^ This behavior may be attributed to the concentration of mechanical stresses along the backbones of pre‐aligned polymer chains oriented along the stretching axis in the electrospun fibers,^[^ ^11^, ^43^, ^45^ ^−^ ^49^ ^]^ as corroborated by the changes in fiber brightness in polarized optical microscopy images recorded at different angles (Figure S2, Supporting Information). In contrast, when randomly oriented fiber mats are stretched, only a fraction of the fibers are deformed along their main axis, resulting in a more gradual, linear increase in mechanical stress. Consequently, aligned fibers exhibited greater stiffness and brittleness, while randomly oriented fibers displayed increased ductility and strain at break (Table S2, Supporting Information).

**Figure 4:**
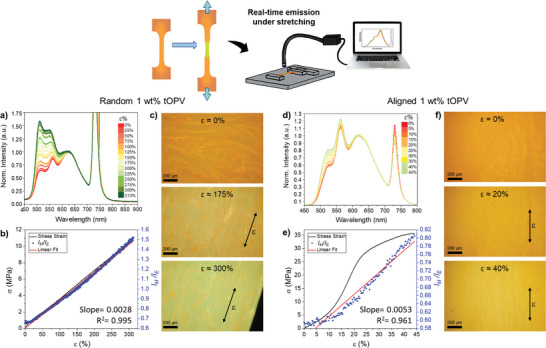
Real‐time emission spectra acquired under stretching of random (a) and aligned (d) PBA‐PU/tOPV 1 wt% fiber mats at the indicated applied strain levels, normalized to the excimer emission band at 630 nm. The symmetric band at 730 nm is due to the excitation beam (365 nm) passing through the grating at twice the wavelength. Plots of the corresponding *I*_M_/*I*_E_ ratio (right *y*‐axis) and mechanical stress (left *y*‐axis) as a function of strain for random (b) and aligned (e) fibers, as determined from in situ mechanofluorescence testing. Fluorescence microscopy images (10× magnification, *λ*_ex_ = 345 nm, reflectance mode, RGB color camera) of random (c) and aligned (f) PBA‐PU/tOPV 1 wt% mats stretched to the indicated strain values and along the specified directions (scale bar = 200 µm).

Having established the morphology and mechanical properties of the fiber mats, we subjected them to uniaxial tensile deformation while simultaneously monitoring their fluorescence in real time (**Figure** **4**; Figure S3, Supporting Information). Notably, all mats displayed a continuous, strain‐dependent increase in *I*_M_/*I*_E_ until sample failure. Specifically, a linear correlation between *I*_M_/*I*_E_ and mechanical strain is found in all samples. However, at strains below ca. 10% a slow increase of *I*_M_/*I*_E_ is observed, overlapping with a non‐linear region in the corresponding stress‐strain curves, a phenomenon also reported in other electrospun materials.^[^ ^50^, ^51^ ^]^ The most pronounced mechanofluorescence was observed for random PBA‐PU/tOPV 1 wt% mats (Figure 4a), for which *I*_M_/*I*_E_ increased from 0.65 to 1.5 upon stretching from 0% to 300% strain (Figure 4b). Above ca. 150% strain, tOPV monomer emission dominates the fluorescence spectra, as is readily apparent when monitoring the fiber mats under the fluorescence microscope (Figure 4c and Video S2, Supporting Information). A change in fluorescence color from orange to yellow is also observed for aligned PBA‐PU 1 wt% fiber mats upon sample failure at ca. 50% strain (Figure 4f and Video S3, Supporting Information).

In the case of aligned fiber mats (PBA‐PU/tOPV 1 wt%), the rate of *I*_M_/*I*_E_ change as a function of strain was nearly doubled in comparison to the changes observed for randomly oriented fiber mats (Figure 4b,e). However, the overall change in emission color was less pronounced for the former, since the lower stretchability of the aligned fibers mats only permitted testing up to ca. 50% strain. These results clearly indicate that fibers aligned parallel to the deformation axis experience greater mechanical strain than those oriented obliquely, leading to a more pronounced mechanochromic response of the aligned fibers at low strains. For both the randomly oriented and aligned fibers, increasing the tOPV content from 1 to 2 wt% resulted in a decrease in the initial *I*_M_/*I*_E_ ratio, and the fluorescence color change is less pronounced, perhaps due to an increased internal absorption that predominantly affects the high‐energy monomer emission (Figure S3a,b, Supporting Information).^[^ ^7^ ^]^

**Figure 5:**
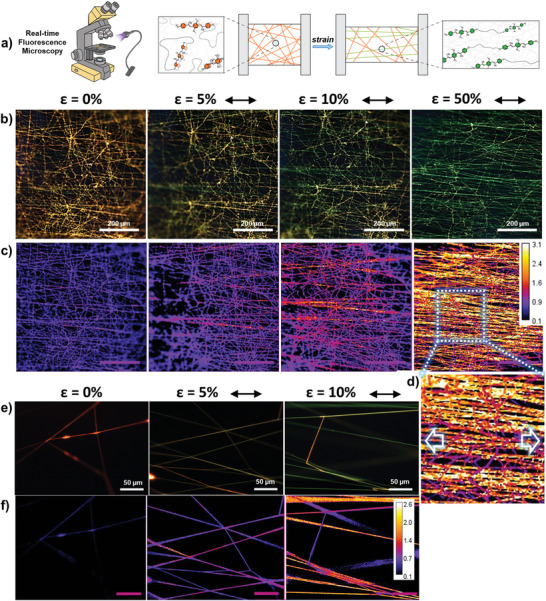
a) Schematic depicting the mechanism that gives rise to mechanochromism in the polymer fibers, as well as the microscopy setup used to measure real‐time fluorescence changes. When deformation is applied, fibers that re‐orient along the stretching direction are subjected to the highest stresses. The force is transmitted to the tOPV additive, which causes the dyes to dissociate and changes the emission from orange (excimeric OPV) to green (monomeric OPV). b,e) Real‐time fluorescence microscopy images (*λ*_ex_ = 345 nm, reflectance mode, RGB color camera) of a thin layer of randomly oriented fibers before deformation (*ε* = 0%) and upon stretching to the indicated strain values, captured at 20× (b) and 50× (e) magnification. Relative stress maps, derived from the ratio of the green and red channel intensities in the RGB images (b) and (e) are shown in (c) and (f), respectively (calibration bars on the upper right). The inset of the stress map at 20× and strain 50% (d) clearly visualizes different stresses acting on fibers oriented parallel (yellow/white fibers, indicating higher stress) or perpendicular (purple/orange fibers, indicating lower stress) to the strain direction. The indicated strain values are calculated relative to the initial distance between the clamps. Scale bars: 200 µm (b, c) and 50 µm (e, f).

Based on the mechanofluorescence properties of the fiber mats, we further investigated the behavior of individual fibers. Leveraging the versatility of the electrospinning technique, we produced ultra‐low density fiber mats on two different devices that enabled the characterization of single fibers or small fiber assemblies during elongation (Figure S4; see Experimental section for details). Fluorescence microscopy images were used to map the local stress distribution in individual fibers based on their orientation. In particular, images of these thin fiber layers acquired during stretching showed that fibers aligned parallel to the stretching axis were the first to exhibit a change in emission color from orange to green (**Figure** **5**a,b), corroborating the increased mechanochromic sensitivity observed in the aligned fiber mats. Moreover, fibers reorienting along the stretching direction displayed a shift from orange to yellow emission (Figure 5b, *ε* = 5 and 10%), ultimately transitioning to green fluorescence with continued stretching (Figure 5b, *ε* = 50%). Analyzing the ratio of the green and red channel intensities in the RGB images enabled the straightforward mapping of the stress distribution within the fibers, with micrometer resolution and millisecond timescale sensitivity, as shown in Figure 5c,d,f. Thus, these microscopy images provide unambiguous evidence that mechanical strain is initially concentrated in fibers pre‐aligned along the stretching axis, while force transfer to oblique fibers induces their gradual alignment. Finally, these observations were further corroborated by high‐magnification fluorescence microscopy imaging of individual fibers, which showed enhanced fluorescence changes for fibers aligned parallel to the stretching axis, while perpendicularly oriented fibers showed little to no change in fluorescence color (Figure 5e,f).

## 3 Conclusion

In summary, we successfully produced homogeneous, bead‐free electrospun nanofibers composed of a semicrystalline polyurethane (PBA‐PU) doped with a mechanofluorescent additive (tOPV). These fibers were used to create non‐woven mats consisting of either randomly oriented or aligned polymeric nanofibers, both of which displayed a fluorescence color change in response to mechanical deformation. Mechanical testing showed that mats with randomly oriented fibers were elastic and ductile, while the aligned fiber mats were stiffer and more brittle. Nevertheless, both types of mats exhibit mechanofluorescence when subjected to tensile deformation, as evidenced by variations in their emission spectra and fluorescence color during stretching. Quantitative in situ monitoring of the fibers’ monomer‐to‐excimer emission intensity ratio (*I*_M_/*I*_E_) reveals that the aligned fiber mats exhibit a higher strain‐dependent increase in *I*_M_/*I*_E_ compared to the randomly oriented mats, indicating that fibers aligned parallel to the stretching direction bear the majority of the applied strain. This finding is further corroborated by fluorescence microscopy imaging of individual fibers, which shows a significantly stronger mechanochromic response for fibers aligned along the deformation axis. Our analysis demonstrates that mechanical stresses are initially absorbed by pre‐aligned fibers, while the remaining fibers only contribute to stress dissipation after reorienting along the stretching axis. Taken together, our findings underscore the critical role of fiber alignment in the mechanochromic response of additive‐doped polymeric fiber mats. By adjusting the fiber orientation, it is in principle possible to tune the mechanochromism of these electrospun mats in terms of both sensitivity and directionality, rendering these materials suitable candidates for precision strain‐sensing applications. Overall, our results demonstrate the potential of electrospun materials as versatile and sensitive strain sensors, paving the way for further research and development in the field of functional materials.

## 4 Experimental Section

### Materials

The mechanofluorescent additive used in this study was a telechelic poly(ethylene‐*co*‐butylene) end‐functionalized with cyano‐subtituted oligo(*p*‐phenylene vinylene) dyes (tOPV; Figure S1a, Supporting Information) and was synthesized as reported by Calvino et al.^[^ ^5^ ^]^ Commercial polyurethane Desmopan 2795A (PBA‐PU, Covestro, *M*_n_ = 93 kDa, *Ð* = 2.1) (Figure S1b, Supporting Information) was employed to produce the electrospun fibers. *N*,*N*‐Dimethylformamide (DMF, ≥99.8%), and tetrahydrofuran (THF, ≥99.9%) were purchased from Sigma Aldrich and used without further purification.

### Sample Preparation

The electrospun nanofiber materials were fabricated using an in‐house electrospinning apparatus consisting of a high‐voltage power supply (Spellman SL b50 P 10/CE/230), a syringe pump (KD Scientific 200 series), and a glass syringe filled with the polymer solution. The syringe was connected to a blunt, stainless‐steel needle (inner diameter = 0.51 mm) through a poly(tetrafluoroethylene) tube. To produce uniaxially aligned fiber mats, a high‐speed rotating collector (length = 120 mm, diameter = 60 mm) with a rotation speed of 5400 rpm (peripheral speed = 17.1 m s^−1^) was employed. This collector facilitated the preferential alignment of the fibers along the direction of drum rotation. Conversely, randomly oriented fiber mats were obtained using a static plate collector. Electrospinning was carried out at room temperature and a relative humidity of 40–50%. PBA‐PU was dissolved in a solvent mixture of THF:DMF (70:30 v v^−1^) at a concentration of 90 mg mL^−1^ together with tOPV at nominal tOPV concentrations of either 1 wt% or 2 wt% in the resulting fibers. The polymer solutions were electrospun using an applied voltage of 18–20 kV, a feed rate of 1.0 mL h^−1^, and a needle‐to‐collector distance of 20 cm. The electrospun mats with different tOPV amounts will henceforth be referred to as PBA‐PU/tOPV X wt%.

### Characterization Methods

Scanning electron microscopy (SEM) analysis was carried out with samples that were sputter‐coated with gold using a Philips 515 SEM at an accelerating voltage of 17 kV. Average fiber diameters and fiber orientations were determined by SEM image analysis of individual fibers (*n* = 300) using ImageJ. The ImageJ Directionality plug‐in, which exploits the local gradient orientation method, was used to determine the orientation of the fibers. For a given fiber mat, the orientation of the individual fibers was averaged across images acquired at four different spots in the sample to accurately determine the overall orientation of fibers in the sample.

A polarized optical microscope (Zeiss Axioscop) was used to observe electrospun fibers at different degrees of rotation with respect to the polarization direction. Fibers were directly collected on glass slides during electrospinning.

Differential scanning calorimetry (DSC) analysis was carried out using a Q2000 DSC apparatus (TA instruments) equipped with a refrigerated cooling system (RCS90) with heating and cooling rates of 10 °C min^−1^. Under nitrogen atmosphere, samples (ca. 6 mg) were placed in a T‐Zero aluminium pan and heated from −90 to 180 °C, cooled to −90 °C, and heated again to 180 °C. Both heating and cooling scans were performed at 10 °C min^−1^. Glass transition temperatures (*T*_g_) were determined at the midpoint of the step‐change in the DSC trace. Melting temperatures (*T*_m_) were determined at the minimum of the most prominent endothermic melting peak.

Mechanical tests were carried out using a Linkam TST350 microtensile stage equipped with a 20 N load cell, controlled by the accompanying Linksys32 software. Dog bone‐shaped specimens (gauge length = 16 mm, width = 5 mm, thickness = 0.05–0.15 mm) were subjected to uniaxial deformation with a strain rate of 30% min^−1^. Mechanical data such as Young's modulus (*E*), strain at break (*ε*_b_), and stress at break (*σ*_b_) were determined from the resulting stress–strain curves.

Solid‐state fluorescence measurements were acquired on an Ocean Optics USB 4000 spectrometer connected to an Ocean Optics LS‐365 LED light source with an excitation wavelength of *λ*_ex_ = 365 nm and an Ocean Optics QR230‐7‐XSR SMA 905 optical fiber. Samples were placed on top of a piece of black paper and the optical fiber was positioned at an angle of approximately 35° relative to the surface of the film at a distance of ≈2 mm. In situ fluorescence data were acquired using Stream Basic software in real‐time while the sample was deformed in the manner described above.

Fluorescence microscopy images were acquired at 20x and 50x magnification using an Olympus BX51 microscope equipped with an Olympus DP72 high‐resolution RGB color camera. The samples were imaged in reflectance mode using an X‐Cite Series 120‐Q Mercury vapor short arc lamp as the excitation source (*λ*_ex_ = 345 nm).

## Conflict of Interest

The authors declare no conflict of interest.

## Supporting information



Supporting Information

Supplemental Video 1

Supplemental Video 2

Supplemental Video 3

## Data Availability

The data that support the findings of this study are available from the corresponding author upon reasonable request.
