# Supplementary material for: In Situ Monitoring of Mechanofluorescence in Polymeric Nanofibers
Source: Macromol Rapid Commun. 2024 Dec 23;46(13):2400855. doi: 10.1002/marc.202400855 (PMC12227233; doi:10.1002/marc.202400855)
Supplement: Supplementary file 1 — Supporting Information [file MARC-46-2400855-s001.docx]

**Supporting Information**

**In-situ monitoring of mechanofluorescence in polymeric nanofibers**

Valentina A. Dini^1^, Derek J. Kiebala^2,3,4^, Damiano Genovese^1,5^, Nelsi Zaccheroni^1,5^, Céline Calvino^2^, Emma Contini^1,5^, Christoph Weder^2,3^, Stephen Schrettl^2,3,6^*, Chiara Gualandi^1,4,7^*

^1^Department of Chemistry “Giacomo Ciamician”, University of Bologna, Via Selmi 2, 40126 Bologna, Italy

^2^Adolphe Merkle Institute (AMI), Polymer Chemistry and Materials, University of Fribourg, Chemin des Verdiers 4, CH-1700 Fribourg Switzerland

^3^National Competence Center in Research Bio-inspired Materials, University of Fribourg, Chemin des Verdiers 4, CH-1700 Fribourg, Switzerland

^4^Department of Chemistry, Johannes Gutenberg University of Mainz, 55128 Mainz, Germany

^5^INSTM UdR of Bologna, University of Bologna, Via Selmi 2, 40126 Bologna, Italy

^6^TUM School of Life Sciences, Technical University of Munich, Maximus-von-Imhof-Forum 2, 85354 Freising, Germany

^7^Interdepartmental Center for Industrial Research on Advanced Applications in Mechanical Engineering and Materials Technology, CIRI-MAM, University of Bologna, Viale Risorgimento, 2, 40136 Bologna, Italy

**1. Characterization of electrospun materials**

**Table S1**. *I*_M_*/I*_E_ (*I_M_* at 520 nm, *I_E_* at 630 nm) values determined by fluorescence spectroscopy and thermal properties determined from the first heating scans of the DSC traces of neat and tOPV-doped samples (*T*_g_ = glass transition temperature determined at the mid-point of the step-change transition; *ΔC_p_* = specific heat capacity; *T*_m_ = melting temperature determined at the minimum of the most prominent endothermic melting peak; *ΔH*_m_ = melting enthalpy).

| **Sample** | ***I_M_/I_E_*** | **T_g_**  **[°C]** | **ΔC_p_**  **[J g^-1^ °C^-1^]** | **T_m_**  **[°C]** | **ΔH_m_**  **[J g^-1^]** |
| --- | --- | --- | --- | --- | --- |
| PBA-PU bulk | n.a. | -47 | 0.22 | 52 | 33.7 |
| PBA-PU fibers^a^ | n.a. | -46 | 0.25 | 53 | 40.4 |
| Random 1% tOPV | 0.65 | -46 | 0.32 | 53 | 34.3 |
| Aligned 1% tOPV | 0.59 | -44 | 0.23 | 52 | 38.5 |
| Random 2% tOPV | 0.19 | -46 | 0.23 | 53 | 35.9 |
| Aligned 2% tOPV | 0.51 | -46 | 0.25 | 53 | 38.8 |
| tOPV bulk | n.a. | -47 | 0.18 | 138 | 44.6 |

a) sample made of aligned fibers.


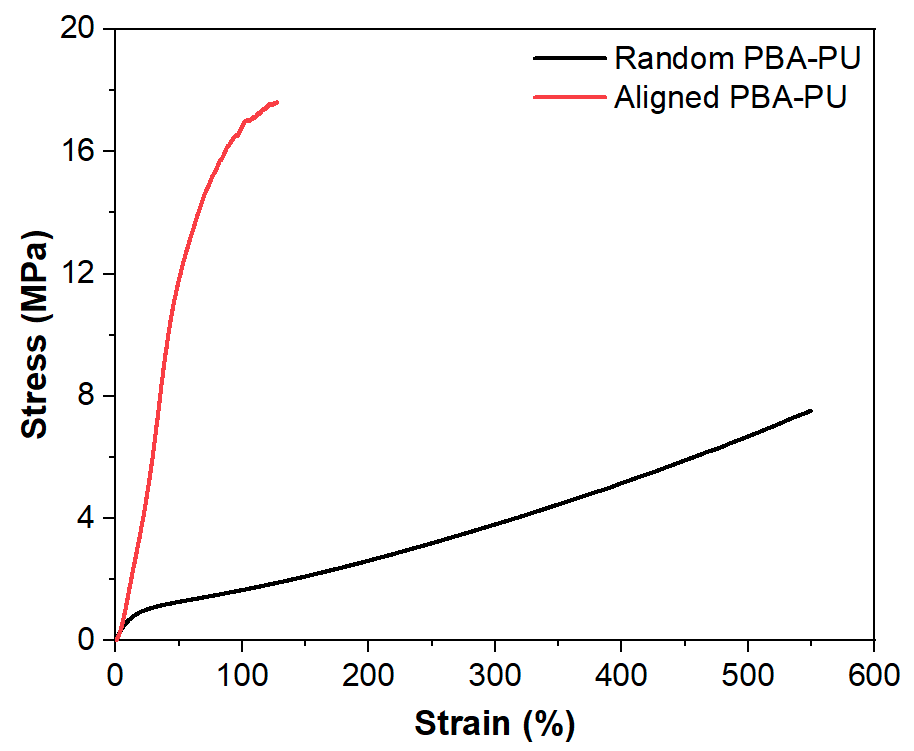


**Figure S1**. Representative stress-strain curves from uniaxial tensile testing (strain rate = 30% min^–1^) of random PBA-PU (black) and aligned PBA-PU (red) fiber mats.

**Table S2**. Young’s modulus (*E*), strain at break (*ε_b_*), stress at break (*σ_b_*), and toughness (*U_T_*) of electrospun PBA-PU/tOPV fiber mats with different fiber orientations and tOPV concentrations. The mechanical properties were determined from the stress-strain curves and the reported values are average and standard deviation of three measurements.

| **Sample** | **E**  **[MPa]** | **ε_b_**  **[%]** | **σ_b_**  **[MPa]** | ***U*_T_**  **[J m^-3^]** |
| --- | --- | --- | --- | --- |
| Random PBA-PU | 5.7 ± 0.6 | 560 ± 30 | 7.2 ± 0.4 | 20 ± 2 |
| Random 1% tOPV | 8 ± 2 | 318 ± 4 | 10 ± 2 | 16 ± 3 |
| Random 2% tOPV | 5.4 ± 0.4 | 230 ± 50 | 6 ± 2 | 5 ± 2 |
| Aligned PBA-PU | 25 ± 2 | 119 ± 9 | 17.3 ± 0.5 | 13 ± 1 |
| Aligned 1% tOPV | 26.1 ± 0.5 | 50 ± 10 | 17 ± 3 | 7 ± 3 |
| Aligned 2% tOPV | 26 ± 3 | 63 ± 3 | 17 ± 1 | 6.4 ± 0.5 |


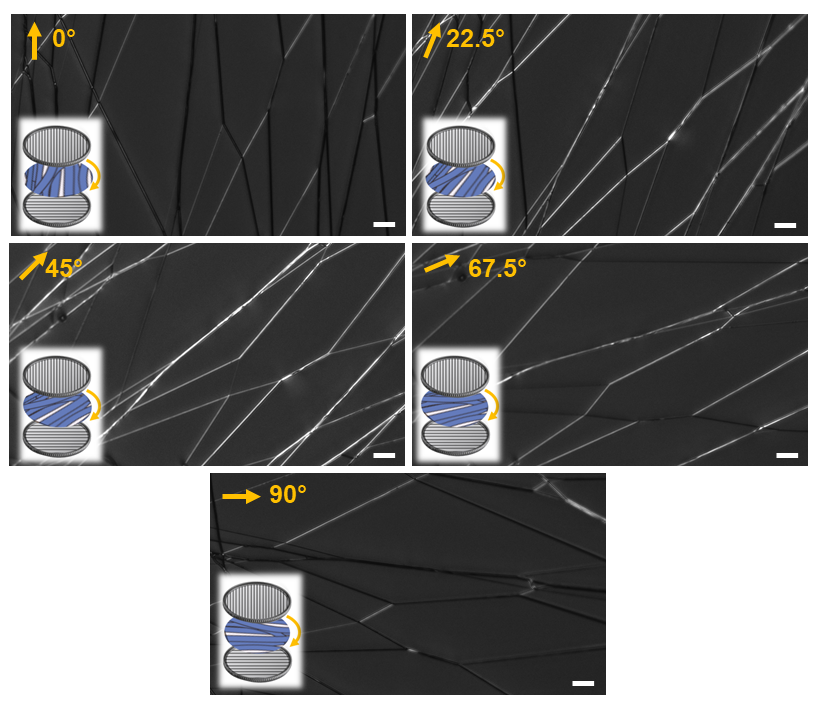


**Figure S2**. Images of aligned electrospun fibers acquired with a polarized optical microscope under different angles with respect to the polarization direction as indicated by the yellow arrows (scale bar = 20 µm).

**2. Mechanochromic characterization of electrospun materials**


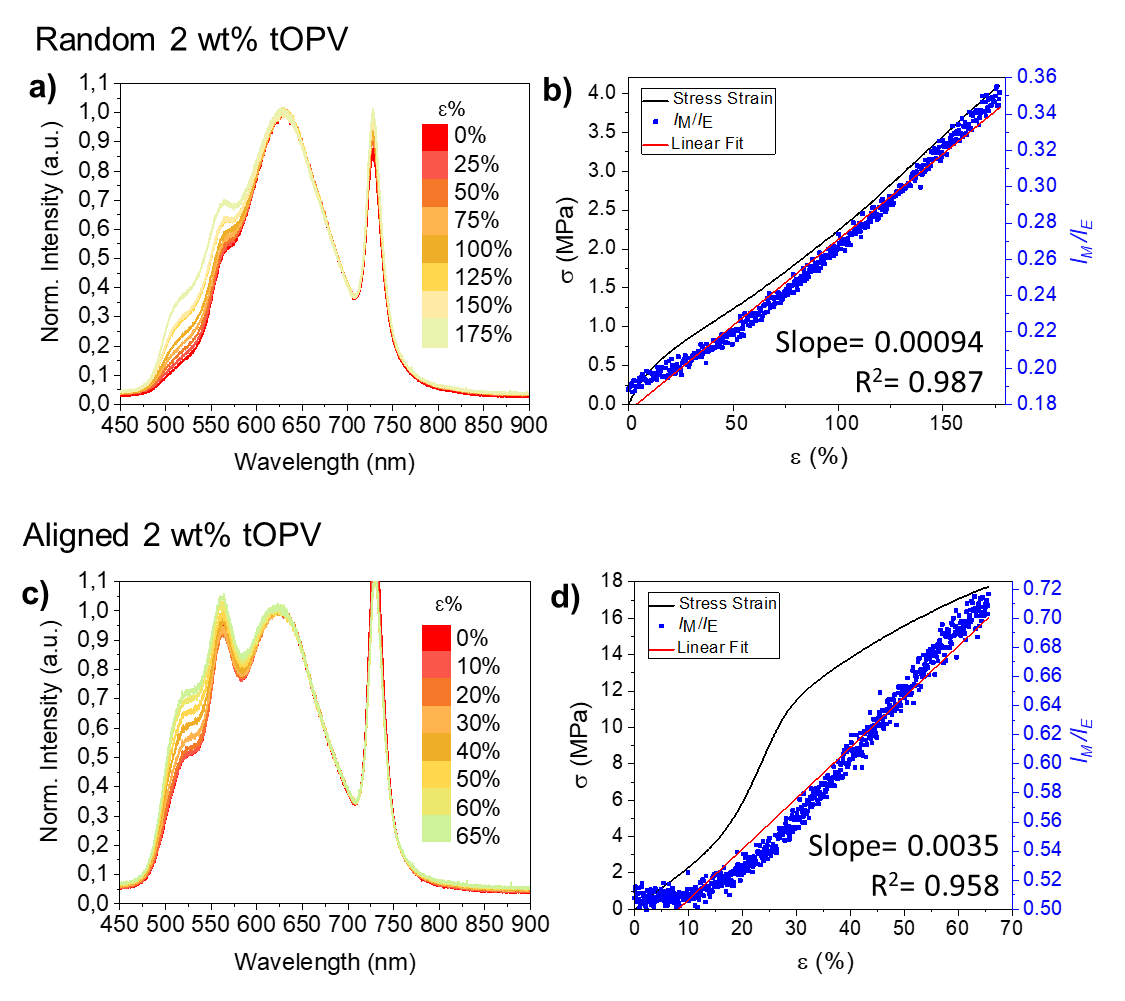


**Figure S3**. a,c) Emission spectra of PBA-PU/tOPV 2 wt% fiber mats with randomly oriented (a) and uniaxially aligned (c) fibers recorded at the indicated applied strains. Spectra are normalized to the excimer emission band at 630 nm. The symmetric band at 730 nm is due to the excitation beam (365 nm) passing through the grating at twice the wavelength. b,d) Plots of the corresponding *I*_M_/*I*_E_ ratio (right y-axis) and mechanical stress (left y-axis) for random (b) and aligned (d) fiber mats as a function of strain.


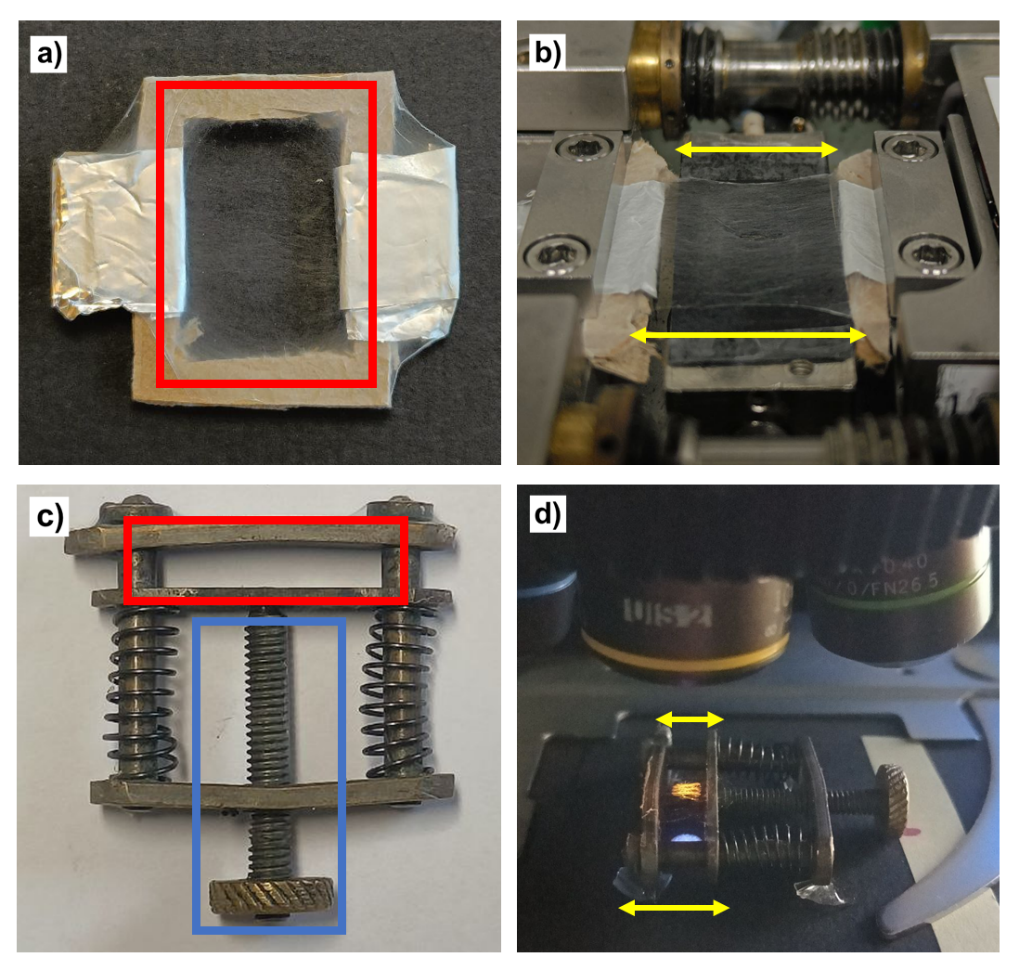


**Figure S4.** Photographs of samples of fiber mats for mechanical testing as well as fluorescence monitoring. a) Photograph of a cardboard mask covered with an ultra-thin layer of electrospun fibers collected in the area indicated in red. b) Photograph of a cardboard mask after fixation in the clamps of the microtensile testing stage. The two sides of the mask parallel to the stretching direction (yellow arrows) were removed after clamping in the stage to exclusively probe the fibers’ mechanical properties during uniaxial deformation. c) Metal brackets onto which a thin layer of fibers was spun in the area indicated in red. The screw (indicated in blue) allows to increase the distance between the brackets, thus elongating the fibers. d) Photograph of the bracket shown in (c) after placement in the fluorescence microscope, with the direction of elongation indicated by yellow arrows.

**Video S1**. Electrospinning of a solution of PBA-PU/tOPV 1 wt% in THF:DMF (70:30 v v^–1^) as observed under UV light.

**Video S2.** Stretching of a random mat of PBA-PU/tOPV 1 wt% fibers under UV light at the fluorescence microscope (10x magnification).

**Video S3.** Stretching of an aligned mat of PBA-PU/tOPV 1 wt% fibers under UV light at the fluorescence microscope (10x magnification).
